# Covariance of maximum likelihood evolutionary distances between sequences aligned pairwise

**DOI:** 10.1186/1471-2148-8-179

**Published:** 2008-06-23

**Authors:** Christophe Dessimoz, Manuel Gil

**Affiliations:** 1 Department of Computer Science, ETH Zurich, 8092 Zurich, Switzerland; 2 Swiss Institute of Bioinformatics, Switzerland

## Abstract

**Background:**

The estimation of a distance between two biological sequences is a fundamental process in molecular evolution. It is usually performed by maximum likelihood (ML) on characters aligned either pairwise or jointly in a multiple sequence alignment (MSA). Estimators for the covariance of pairs from an MSA are known, but we are not aware of any solution for cases of pairs aligned independently. In large-scale analyses, it may be too costly to compute MSAs every time distances must be compared, and therefore a covariance estimator for distances estimated from pairs aligned independently is desirable. Knowledge of covariances improves any process that compares or combines distances, such as in generalized least-squares phylogenetic tree building, orthology inference, or lateral gene transfer detection.

**Results:**

In this paper, we introduce an estimator for the covariance of distances from sequences aligned pairwise. Its performance is analyzed through extensive Monte Carlo simulations, and compared to the well-known variance estimator of ML distances. Our covariance estimator can be used together with the ML variance estimator to form covariance matrices.

**Conclusion:**

The estimator performs similarly to the ML variance estimator. In particular, it shows no sign of bias when sequence divergence is below 150 PAM units (i.e. above ~29% expected sequence identity). Above that distance, the covariances tend to be underestimated, but then ML variances are also underestimated.

## Background

The estimation of evolutionary distances between gene/protein sequences is one of the most important problems in molecular evolution. In particular, it lies at the heart of most phylogenetic tree construction methods. The estimation of such distances is a two step process: first, homologous characters are identified, then the distances are estimated from the character substitution patterns. The most accurate matching of homologous characters is obtained by multiple sequence alignments (MSAs). Indeed, by considering all sequences simultaneously, MSAs yield a consistent and in principle optimal grouping of the homologous characters. Unfortunately, MSAs are hard to compute optimally (time complexity exponential in the number of sequences), and thus are in practice computed using heuristics. Alternatively, the sequences can be analyzed exclusively on the basis of pairs of sequences, using an algorithm such as Smith-Waterman [1] that yields optimal pairwise alignments (OPAs). This approach is often taken by large-scale comparative genomics analysis such as MIPS, OMA or RoundUp [2-4], which analyze the sequences pairwise due to computational constraints.

**Figure 1:**
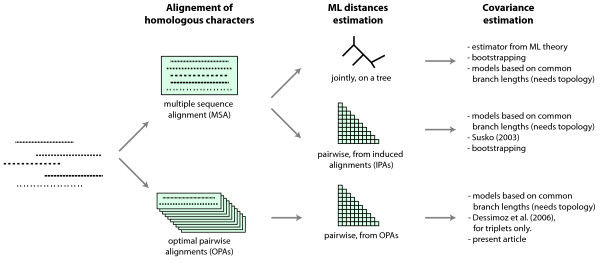
**Overview of approaches to estimate evolutionary distances and their covariances**. A set of *n *sequences can be aligned jointly to obtain an MSA or in a pairwise optimal manner resulting in $\left(\begin{array}{c} n\\ 2 \end{array}\right)$ optimal pairwise alignments (OPAs). Given a hypothesis of character homology, distance estimation per ML can essentially be done in two ways: jointly on a tree or pairwise. In the first case a tree's branch-lengths are estimated simultaneously. This requires an MSA. In the second case pairwise distances are estimated either from MSA induced pairwise alignments (IPAs) or from the OPAs. The distance estimators are afflicted with an error expressed by their variances and covariances. In all cases, the covariances can be modeled as a function of shared branch lengths, but this requires a phylogenetic tree. When distances are estimated based on an MSA, the variances and covariances can be obtained from ML theory or by bootstrapping over the MSA's columns. In the case of OPAs, these techniques cannot be directly applied (see *Methods*). We have previously presented a covariance estimator for the case where the two OPAs in question share a sequence (i.e. for triplets). In this paper, we introduce an estimator for the general case.

Once the homologous characters are identified, the second step of distance estimation can proceed. The method of choice is a maximum likelihood (ML) estimation based on some model of evolution. There too, the distances can either be estimated simultaneously from all sequences using a combination of tree topology inference and joint optimization of all branches, or pairwise, by estimating the distances between every pair of sequences. Joint estimation requires MSAs, while pairwise distance estimation can be done from either OPAs or from the pairwise alignments induced by an MSA (IPAs). Fig. 1 provides an overview of the different approaches.

In all cases, the estimation of evolutionary distances is subject to inference uncertainty, which is commonly quantified by their variances and covariances. Indeed, the distance variance information can be used to build confidence intervals around the estimate; covariances of pairs of distances can be used to build the confidence intervals of combinations of distances. Examples of applications include generalized least squares (GLS) phylogenetic tree building [5] construction of confidence sets of trees [6], test for monophyly using likelihood ratios [7], comparison of evolutionary distances for orthology inference [3], or distance-based lateral gene transfer detection [8]

Variance estimates are provided by ML theory in both joint and pairwise distances estimation. However, ML theory only provides covariance estimates if all distances are estimated jointly. Covariance estimates for distances computed from IPAs in the context of specific parametric substitution models have been reported by Hasegawa et al. [9] and Bulmer [6], and were generalized by Susko [10] to all Markovian models of evolution. Furthermore, the covariance of distances from IPAs can also be estimated (though much more slowly) through bootstrapping [11]. As for the covariance of distances obtained from OPAs, the main difficulty in computing them is that, since sequence pairs are aligned individually, they usually have inconsistencies in their inference of the homologous characters (or else, computing an MSA from pairwise alignments would be trivial). Thus, the alignments cannot be partitioned in consistent "columns" of characters, and neither Susko's method nor resampling approaches such as bootstrapping can be applied. Indeed, in the case of analyses relying exclusively on pairwise comparison and distance estimation, i.e. where no MSA computation can be afforded, we are not aware of any previously published estimator for the covariance of distances estimates from pairwise alignments.

We have shown in a previous article [12] a numerical approximation for the constrained case of the covariance of two OPA distances involving a common sequence (i.e. on a triplet of sequences), for empirical substitution models such as PAM or JTT. In this article, we present an estimator for the covariance of ML distances estimated from OPAs that works on triplets and quartets of sequences. This solves the problem of sets of sequences of arbitrary size, because each covariance involves at most four sequences at a time. Thus, the full covariance matrix is naturally obtained through quartet analysis. We analyze the performances of the estimator in terms of bias and variance. Finally, we compare the results obtained on triplets of sequences to our previous work.

## Results and Discussion

**Figure 2:**
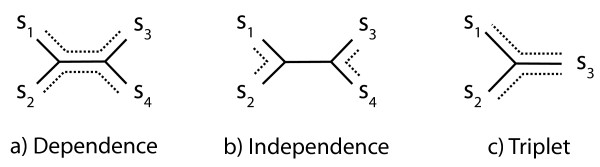
**Possible topological relations of sequences**. For two pairwise distances, one can distinguish three possible underlying topological configurations relating them. If they are estimated between four sequences, there are two possible configurations. Either they share some common evolution (a) or they are independent (b). In the third configuration, the two distances are estimated from two OPAs that share a sequence (c).

In the following, we present and analyze the performances of the estimator for the covariance of two distances. For this purpose, it is informative to analyze the results separately for the following three different underlying topological relations, illustrated in Fig. 2:

### Case of dependence

The two distances are estimated between four distinct sequences, and they have some evolution in common (i.e. the two distance involve a common branch on the tree). With such an evolutionary history, the two distances estimates covary positively.

### Case of independence

The two distances are estimated between four distinct sequences, but they have no evolution in common (i.e. the two distance involve distinct branches on the tree). This case is informative, because a central assumption in most evolutionary models is that evolution on different branches is independent [13]. With no branch in common, the distances should not covary [6]. Thus, such a topology can be used to test the estimators as negative control.

### Case of triplet

The two distances involve a common sequence, and have some evolution in common. This case is of special interest, because we have previously presented an alternate estimator for this particular case using a different approach [12]. Thus, we can compare our results to this approach, hereafter called "the numerical approximation".

Note that the covariances are estimated using the same algorithm in all three cases: we only distinguish them from each another for the purpose of this analysis.

To assess the performance of the covariance estimator, it was compared against the Monte Carlo covariance estimator. In short, each point shown in the figures was obtained from 40,000 sets of sequences mutated along a random quartet subtree of the tree of life (see *Methods *below). That way, the evaluation is based on tree samples that are distributed as closely as possible to real biological data. To account for gene families with varying rates, each quartet was scaled with a random factor uniformly distributed between 0.5 and 2. Note that results corresponding to very large distance constitute extreme cases; for instance, when sequences are 150 PAM units apart, each position has, on average, mutated 1.5 times.

**Figure 3:**
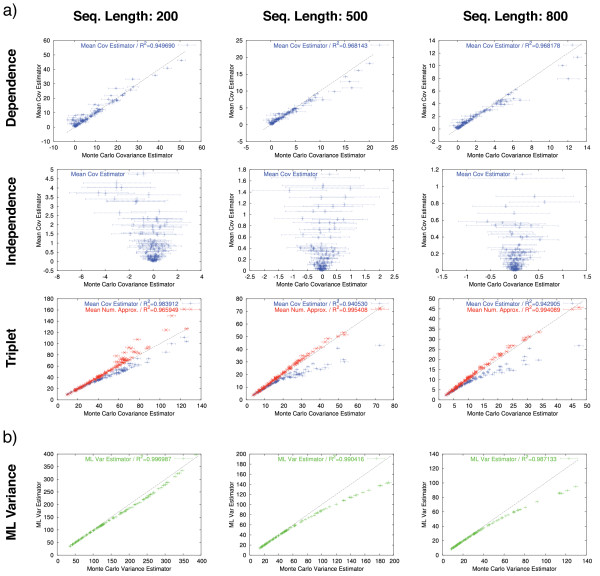
**Comparison of the covariance estimator and the ML variance estimator with their Monte Carlo counterparts**. Error-bars indicate 95% confidence intervals. **a) **Monte Carlo covariance estimator vs. average of the covariance estimator for sequence lengths of {200, 500, 800} AA. In the dependence case, the estimator appears unbiased in most cases. In the independence case, the estimator shows a slight upward bias, but the absolute values are close to zero. In the triplet case, a downward bias with increasing covariance is visible. **b) **Monte Carlo variance estimator vs. average of ML variance estimator. A downward bias with increasing variance is visible.

Fig. 3a shows the mean of our estimator versus the Monte Carlo estimator in nine scatterplots arising from combining the topologies mentioned above (rows) with three different sequence lengths (columns). In the case of dependence, the first row, we see that our estimator lies in about 80% of the cases within the 95% confidence interval of the Monte Carlo estimator. In the case of independence, both estimators are close to zero, though our estimator shows a minor upward bias in some cases. The third row gives the result of both the covariance estimator introduced here, as well as the numerical approximation from our previous study [12]. Here, we see that though the former performs well in cases of lower covariance values, it shows a clear downward bias in cases of larger covariances. The numerical approximation does not present any apparent sign of bias, which is hardly surprising, given that it was obtained through regression. What is however surprising, is that, given its simple structure, it performs better than the covariance estimator, which takes into account more data and is backed by a more detailed model.

It is instructive to compare the absolute bias of the covariance estimator to the well-known ML variance estimator (see e.g. [14]). As can be seen in Fig. 3b, the ML variance is also biased for high variance values. We conjecture that this is mainly due to mis-aligned positions, which cause model violations in the parameter estimation. This problem is also likely to affect the covariance estimator. Even more directly, the ML variance estimator is a factor in the expression of the covariance estimator (see *Methods*), so any error in the ML variance is propagated to the covariance estimator. At this point, improving the ML estimator for cases of high divergence is likely to require better alignments, or an explicit modeling of the mis-aligned positions, which is beyond the scope of the present work.

**Figure 4:**
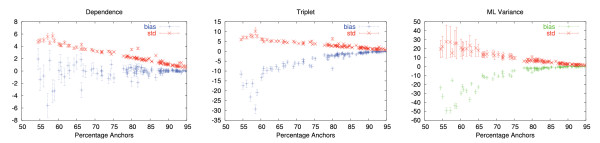
**Bias and standard deviation of the covariance and ML variance estimators**. Average percentage of anchors vs. bias and standard deviation of the covariance estimator for sequence length of 500 AA. Error-bars indicate the 95% confidence intervals. The bias increases with decreasing fraction of anchors. The bias is smaller than the standard deviation when percentage of anchors is greater than 65% (dependence), 80% (triplet) and 75% (ML variance).

**Figure 5:**
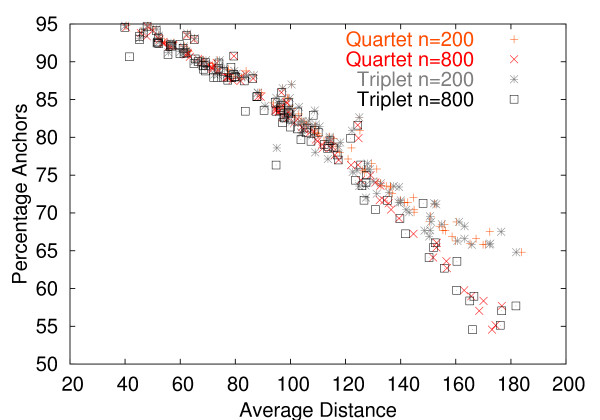
**Relation between distance and percentage of anchors**. Horizontal axis: Average of the two distances for which the covariance has been estimated. Vertical axis: Average percentage of anchors. The *Quartet *labels refer to the dependence case. The fraction of anchors decreases with increasing distance.

Further, to put the bias of the covariance estimator into perspective, we compared it to the standard deviation of the estimator. Fig. 4 presents the bias and standard deviation as function of the average number of anchors for sequence length of 500. The anchors are the positions that are consistently aligned in the OPAs involved (see methods for the precise definition). Both bias and standard deviation strongly depend on the fraction of anchors, which can be thought of as a measure of alignment quality. Fig. 5 depicts the dependency between percentage of anchors and average distance. As one would expect, the fraction of anchors decreases as divergence increases. For a fraction of anchor positions below 60%, the average of the two distances involved in the covariance computation is always greater than 150 PAM. In Fig. 4, we first consider the bias and standard deviation for the case of dependence. When the fraction of anchor positions is above 60% (this is the case for approximately 85% of the quartets of sequences in families of orthologs in OMA [3], data not shown), the bias is far smaller than the standard deviation, and is therefore likely to have little negative impact in practice.

In the case of triplets, the bias exceeds the standard deviation already when the fraction of anchors is about 80%. The ML variance estimator has this transition around 75% of anchors. In the case of independence, where we expect our covariance estimator to be zero, its bias is always much smaller than its standard deviation (data not shown).

**Figure 6:**
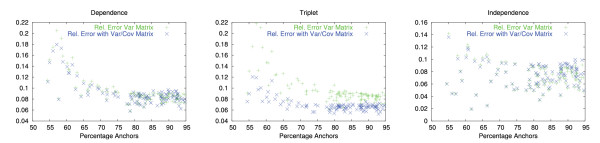
**Relative error of covariance matrix**. Average relative error of variance matrices and variance/covariance matrices for a sequence length of 500 AA. Dependence and independence cases: Variance matrices and variance-covariance matrices have comparable error. Triplet case: Variance-covariance matrices have lower error.

Most applications of the covariance estimator involve the covariance matrix. Let $\hat{A}$ be an approximation to the matrix *A*. We refer to $\frac{||\hat{A}-A|{|}_{2}}{||A|{|}_{2}}$ as the relative error in $\hat{A}$, where ||·||_2 _denotes the two-norm. Fig. 6 shows the relative error of the 2 × 2 variance-covariance matrices computed with the ML variance estimator in the diagonal entries and our covariance estimator in the off-diagonal entries, and the same 2 × 2 matrices with only diagonal entries. The plots show that for the dependence case the the matrices with both covariance and ML variance estimators have a equal or lower relative error than the matrices with the ML variance only, except for a few cases in the region with a high fraction of anchors. In the triplet case, the variance-covariance matrices have always lower error then variance matrices. Finally, in the case of independence, the matrices with covariance do not always have lower relative error, but this is expected, because the true covariance is null in this special case.

## Conclusion

We have presented a method to estimate the covariances of distances estimated from pairwise alignments. It does not require the construction of MSAs, which are hard to compute and therefore are only approximated in practice. Furthermore, it does not rely on phylogenetic trees as it is the case with covariance estimation from joint ML, or in covariance estimation methods that model the covariances as a function of shared branch lengths [15,16]. Tree building is not only a costly process, but is also subject to inference errors.

The accuracy of our estimator is comparable to the ML variance estimator. Both estimators are biased but in both cases the bias is, for distances below 150 PAM, far smaller than their standard deviation. The bias of the covariance estimator (as well as the ML variance, and to some extent the distance estimators) becomes worse with declining percentage of anchors. These biases arise because the alignment positions under scrutiny do not constitute an unbiased subsample of the true homologous positions. Note that misaligned positions are likely to affect distances from MSAs too. A solution to this problem would lead to better distance estimates in the first place. In the meanwhile, it is probably best to issue a warning if the percentage of anchors falls below some threshold.

The estimation of evolutionary distances is a very important process in molecular evolution, and therefore the covariance estimator presented here will be of use for various applications, such as the construction of GLS trees on OPA distances, the construction of confidence sets of trees based on the GLS test statistic, relative-rate tests, distance-based lateral gene transfer detection, and in general in any process that needs to estimate confidence of distance combinations.

## Methods

### Covariance of distances from OPAs

In this section we derive a covariance estimator for ML distances from OPAs.

#### Preliminaries

**Figure 7:**
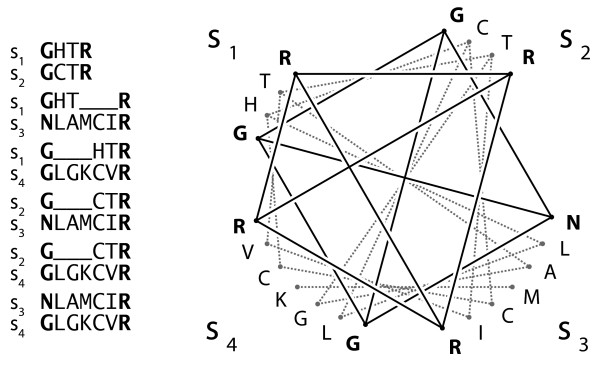
**Example of anchors**. The six pairwise alignments of four example sequences (left) and the corresponding graph-representation (right). The consistent positions are in bold.

The columns of an MSA are a consistent hypothesis of character homology for a set of sequences. With OPAs on the other hand, we have the problem that for a set sequences, the resulting pairwise alignments are not always consistent in their inference of the homologous characters. Fig. 7 depicts an example. Let *s*_*i*,*j *_be the character at position *j *in a sequence *s*_*i*_. Only characters in bold, for example {*s*_1,1_, *s*_2,1_, *s*_3,1_, *s*_4,1_}, are consistently aligned in the OPAs. We call such a consistent set of characters an *anchor*. On the other hand, *s*_1,2 _is aligned to *s*_2,2 _and to *s*_3,2_, so in a consistent situation it would follow that *s*_2,2 _and *s*_3,2 _should be aligned, but it is not the case.

Given *m *sequences, the anchors can formally be defined as follows: Define a graph *G*({*s*_*i*_}) with $\sum_{i}^{m}\left|s_{i}\right|$ vertices labeled by *s*_*i*,*j *_. We join vertices $s_{i_{1},j_{1}}$ and $s_{i_{2},j_{2}}$ if the corresponding characters are aligned in the $\text{OPA}(s_{i_{1},}s_{i_{2}})$. The set of anchors for the $\left(\begin{array}{c} m\\ 2 \end{array}\right)$ OPAs is defined as the set of all cliques of size $\left(\begin{array}{c} m\\ 2 \end{array}\right)$ in *G*({*s*_*i*_}). By construction, the sub-alignments induced by the anchors define an MSA. In the derivation of our covariance estimator, we assume that the anchor positions are correctly aligned. For the non-anchor positions, we know that some proportion is wrongly aligned in at least one of the $\left(\begin{array}{c} m\\ 2 \end{array}\right)$ OPAs. We do not know, though, which positions and in which alignments. In this paper we are interested in the covariance of distances from two OPAs. In each case the anchors are determined from the particular sequences involved in the corresponding covariance estimation. If the two OPAs share a sequence *m *= 3, otherwise *m *= 4. The following pseudocode shows how the anchors can be found for *m *= 4. It uses a function $\text{M}(s_{i_{1}},s_{i_{2}},j_{1})$ which returns the index *j*_2 _of the character $s_{i_{2},j_{2}}$ of *s*_*i*2 _aligned to $s_{i_{1},j_{1}}$ in $\text{OPA}(s_{i_{1},}s_{i_{2}})$.

Anchors ← {}

**for ***j*_1 _← 1 **to ***length*(*s*_1_) **do**

   *j*_2 _← M(*s*_1_, *s*_2_, *j*_1_); *j*_3 _← M(*s*_1_, *s*_3_, *j*_1_); *j*_4 _← M(*s*_1_, *s*_4_, *j*_1_)

   **if **M(*s*_2_, *s*_3_, *i*_2_) = *j*_3 _**and **M(*s*_2_, *s*_4_, *i*_2_) = *j*_4 _**and **M(*s*_3_, *s*_4_, *i*_3_) = *j*_4 _**then**

      Anchors ← Anchors ∪ $\left\{[s_{1,j_{1}},s_{2,j_{2}},s_{3,j_{3}},s_{4,j_{4}}]\right\}$

end

end

#### Formulation of the covariance estimator

Let *p*(*X*_*j*_, *d*) denote the probability of a homologous character-pair *X*_*j *_for the *j*-th OPA when the distance is taken to be *d*. We assume that the gap-positions have been removed from the alignments and that the *j*-th OPA has length *n*_*j *_. Denote ${\hat{d}}_{j}$ the distance obtained by ML and *d*_*j *_the true distance. It is well known from ML theory (see e.g. [14]) that under appropriate smoothness conditions, the variance of ${\hat{d}}_{j}$ is

(1) $$V_{j}=\frac{1}{n_{j}}{\left(E\left[-\frac{\partial^{2}}{\partial d_{j}^{2}}\ln(p(X_{j},d_{j}))\right]\right)}^{-1}.$$

Let the score function for the *j*-th OPA be

(2) $$u_{j}(d)=\sum_{l=1}^{n_{j}}\frac{\partial }{\partial d}\ln(p(x_{j,l},d)),$$

where *x*_*j*,*l *_is the realization of *X*_*j *_at position *l*. To abbreviate, we set $u_{j,l}(d)=\frac{\partial }{\partial d}\ln(p(x_{j,l},d))$. As mentioned by Susko [10], ML results yield

(3) $$\sqrt{n_{j}}({\hat{d}}_{j}-d_{j})=-\sqrt{n_{j}}V_{j}u_{j}(d_{j})+o_{p}(1).$$

Based on equation (3) we derive now an expression for the covariance of two distance estimates ${\hat{d}}_{j}$ and ${\hat{d}}_{k}$. Along this paper, variables with a superscript *A *refer to anchors, *N *refer to non-anchors. Since virtually all Markovian models of evolution assume independent positions, we can split the score functions in a part corresponding to the anchor positions and a non-anchor part:

(4) $$u_{j}(d)=u_{j}^{A}(d)+u_{j}^{N}(d).$$

We assume that the sums in $u_{j}^{A}(d)$ and $u_{k}^{A}(d)$ are ordered such that $u_{j,l}^{A}(d)$ and $u_{k,m}^{A}(d)$ are part of the same anchor iff *l *= *m*. Since, up to high order terms, (${\hat{d}}_{j}$ - *d*_*j*_) is equal to *-V*_*j*_*u*_*j *_(*d*_*j*_) we can write for the covariance of ${\hat{d}}_{j}$ and ${\hat{d}}_{k}$

(5) $$cov\left({\hat{d}}_{j},{\hat{d}}_{k}\right)=cov\left(\left({\hat{d}}_{j}-d_{j}),({\hat{d}}_{k}-d_{k}\right)\right)\approx$$

(6) $$cov\left(-V_{j}\{u_{j}^{A}(d_{j})+u_{j}^{N}(d_{j})\},-V_{k}\{u_{k}^{A}(d_{k})+u_{k}^{N}(d_{k})\}\right)$$

(7) $$=V_{j}V_{k}\{cov(u_{j}^{A}(d_{j}),u_{k}^{A}(d_{k}))+cov(u_{j}^{N}(d_{j}),u_{k}^{N}(d_{k}))+cov(u_{j}^{A}(d_{j}),u_{k}^{N}(d_{k}))+cov(u_{j}^{N}(d_{j}),u_{k}^{A}(d_{k}))\}.$$

Correlations between distance arise from common mutation events (on common branches on the "true" tree). As mentioned above, positions in a sequence are stochastically independent from one another. We assume that the anchors are correctly aligned. Consequently, characters in the anchor and non-anchor parts cannot be homologous to each other. Therefore $cov(u_{j}^{A}(d_{j}),u_{k}^{N}(d_{k}))$ and $cov(u_{j}^{N}(d_{j}),u_{k}^{A}(d_{k}))$ are both zero. The expression becomes

(8) $$V_{j}V_{k}\{cov(u_{j}^{A}(d_{j}),u_{k}^{A}(d_{k}))+cov(u_{j}^{N}(d_{j}),u_{k}^{N}(d_{k}))\}$$

(9) $$=V_{j}V_{k}\left\{cov\left(\sum_{l=1}^{n_{A}}u_{j,l}^{A}(d_{j}),\sum_{m=1}^{n_{A}}u_{k,m}^{A}(d_{k})\right)+cov\left(\sum_{l=1}^{n_{j}-n_{A}}u_{j,l}^{N}(d_{j}),\sum_{m=1}^{n_{k}-n_{A}}u_{k,m}^{N}(d_{k})\right)\right\}$$

(10) $$=V_{j}V_{k}\left\{\left(\sum_{l=1}^{n_{A}}\sum_{m=1}^{n_{A}}cov\left(u_{j,l}^{A}(d_{j}),u_{k,m}^{A}(d_{k})\right)+\sum_{l=1}^{n_{j}-n_{A}}\sum_{m=1}^{n_{k}-n_{A}}cov\left(u_{j,l}^{N}(d_{j}),u_{k,m}^{N}(d_{k})\right)\right)\right\},$$

where *n*_*A *_is the number of anchors. Because of the correctness assumption of the anchors, all pairs that are not part of the same anchor are non-homologous, and therefore, their covariance is zero, i.e. for *l *≠ *m*, $cov\left(u_{j,l}^{A}(d_{j}),u_{k,m}^{A}(d_{k})\right)=0$ and we get

(11) $$V_{j}V_{k}\left\{\sum_{l=1}^{n_{A}}cov\left(u_{j,l}^{A}(d_{j}),u_{k,l}^{A}(d_{k})\right)+\sum_{l=1}^{n_{j}-n_{A}}\sum_{m=1}^{n_{k}-n_{A}}cov\left(u_{j,l}^{N}(d_{j}),u_{k,m}^{N}(d_{k})\right)\right\}.$$

We assumes that the $u_{j,l}^{A}(d_{j})$ are i.i.d. We denote the corresponding random variables $U_{j}^{A}$. The assumption is justified due to the Markov model and the correctness assumption of the anchors. As to the $u_{j,l}^{N}(d_{j})$ some proportion may be homologous, but we do not know which one. Determining the homologous pairs would solve the problem of MSA construction (known to be hard and not our goal here). Instead, we take the working assumption that the $u_{j,l}^{N}(d_{j})$ and $u_{k,m}^{N}(d_{k})$ do not covary. With the two assumptions the expression of the covariance approximation becomes:

(12) $$cov\left({\hat{d}}_{j},d_{k}\right)\approx V_{j}V_{k}n_{A}cov\left(U_{j}^{A},U_{k}^{A}\right).$$

By using the form of equation (12), we obtain an estimator for the covariance. The variance *V*_*j *_is estimated by

(13) $${\hat{V}}_{j}=\frac{1}{n_{j}}{\left(\frac{1}{n_{j}}\sum_{l=1}^{n_{j}}-\frac{\partial^{2}}{\partial d_{j}^{2}}\ln\left(p(x_{j,l},{\hat{d}}_{j})\right)\right)}^{-1}.$$

The estimate for the covariance of the anchor part is the well-known unbiased estimator

(14) $$\hat{cov}(U_{j}^{A},U_{k}^{A})=\frac{1}{n_{A}-1}\sum_{l=1}^{n_{A}}\left(u_{j,l}^{A}({\hat{d}}_{j})-{\bar{u}}_{j}^{A}({\hat{d}}_{j}))(u_{k,l}^{A}({\hat{d}}_{k})-{\bar{u}}_{k}^{A}({\hat{d}}_{k})\right),$$

where ${\bar{u}}_{j}^{A}$ denotes the sample mean.

### Simulation methods

To evaluate the performance of the covariance estimator we performed a Monte Carlo simulation on quartets and compared our estimator to the sample covariance (also referred to as the Monte Carlo covariance).

#### Sampling of quartets

The quartets were sampled uniformly from a variance weighted least squares (WLS) tree on 352 species. The WLS tree was inferred by the *LeastSquaresTree *function in Darwin [17]. To obtain the input distance and variance matrices for *LeastSquaresTree *we used data from the OMA project [3]. The inter-species distances were determined as average PAM distances over sets of groups of orthologs. A total of 100 quartets were sampled, each one contributing one data-point to the plots shown here.

#### Simulation procedure for one quartet

To explore the branch-length space, while preserving the relative branch-length structure given by the WLS tree we applied an uniformly distributed U(0.5,2) expansion/contraction factor on each quartet. Then, we generated 40,000 times three random sequences of length *m *= {200, 500, 800} and mutated each of them along the dilated model quartet. We assumed a Markovian model of evolution using the updated PAM matrices [18] and introduced gaps of Zipfian distributed length [19].

We applied our covariance estimator on each of the 40,000 quartets and estimated its expected value and variance to compare it against the sample covariance which we also refer to as *Monte Carlo covariance*. In the analysis of the results, we treated the sample covariance as a reference value, as it constitutes an unbiased estimator for the true covariance. The biases reported in the result section are defined as the estimate of the expected value of our covariance estimator minus the Monte Carlo covariance. Note that being an estimator itself, the sample covariance's variance had also to be taken into account in the analysis of the results.

## Authors' contributions

CD and MG equally contributed to ideas, execution and writing.
